# The Role of Attachment in Body Weight and Weight Loss in Bariatric Patients

**DOI:** 10.1007/s11695-017-2796-1

**Published:** 2017-07-06

**Authors:** Abigail Nancarrow, Amelia Hollywood, Jane Ogden, Majid Hashemi

**Affiliations:** 1 Department of Clinical Health Psychology and Neuropsychology, St Georges University Hospital NHS Foundation Trust, London, UK; 2 Health Services Research School of Pharmacy, Whiteknights, PO Box 226, Reading, RG6 6AP UK; 3 Department of Psychology, University of Surrey, Guildford, GU2 7XH UK; 4 GI Services, University College London Hospital, Ground Floor West, 250 Euston Road, London, NW1 2PG UK

**Keywords:** Attachment, Obesity, Weight gain, Weight loss

## Abstract

**Purpose:**

The aim of this study is to explore the role of attachment styles in obesity.

**Material and Methods:**

The present study explored differences in insecure attachment styles between an obese sample waiting for bariatric surgery (*n* = 195) and an age, sex and height matched normal weight control group (*n* = 195). It then explored the role of attachment styles in predicting change in BMI 1 year post bariatric surgery (*n* = 143)***.***

**Results:**

The bariatric group reported significantly higher levels of anxious attachment and lower levels of avoidant attachment than the control non-obese group. Baseline attachment styles did not, however, predict change in BMI post surgery.

**Conclusion:**

Attachment style is different in those that are already obese from those who are not. Attachment was not related to weight loss post surgery.

## Introduction

Obesity is recognised as a multifactorial problem resulting from the obesogenic environment, genetics, nutrition and physical activity. Research has also indicated a role for the impact of family dynamics on body weight, with a particular focus on attachment. Attachment theory suggests that infants are born with a range of innate behaviours to maximise their chance of survival and that whilst exploratory behaviour enables the infant to explore their social world, attachment behaviour draws others towards them in a time of need or distress [1]. It is also argued that these behaviours influence the development of the psychological self and the individual’s ability to understand themselves in relation to others within the context of their social world [2, 3]. Attachment styles are described as ‘patterns of interpersonal interactions and affect regulation in adulthood that describe how individuals cope with distress and perceive others’ [4] and reflect the ways in which people interact with each other and regulate their emotions [1–4]. Attachment styles can be classified as secure or insecure [1, 2]. Secure attachment is characterised by a person having a positive view of themselves and others, positive expectations about others’ availability, the ability to express and share emotions, to adaptively regulate affect and to use constructive means of coping [2, 5]. According to attachment theory, it is essential for infants to form a ‘secure’ attachment with their caregiver so they can feel protected and safe whilst engaging with others as a means to learn about their role within the relationship and to make sense of their own and others’ psychological nature [2, 6]. It is also emphasised that secure attachments are necessary for affect regulation and the individuals’ ability to regulate their emotions in order to maintain a state of wellbeing [3]. In contrast, insecure attachment is more problematic and can be subdivided into ‘avoidant’ and ‘anxious’ subtypes. Someone with an avoidant attachment style may develop a positive view of themselves but a negative view of others, have difficulty expressing emotions and a preference for emotional distance from others [2]. Adults with anxious attachment styles however, may have a negative view of themselves but a positive view of others with overactive emotional systems and a need for closeness [2]. Attachment styles have been shown to be closely linked with emotional regulation and specifically the use of emotional eating as a coping mechanism [5]. In particular, children whose early caregiver is unreliable and unresponsive during times of need may well turn to food as a coping mechanism in the absence of strategies to manage their emotions [2].

To date, the majority of research exploring links between attachment and eating behaviour has focused on eating disorders including anorexia nervosa, bulimia nervosa and binge eating disorder [7, 8] with research consistently indicating that eating psychopathology is associated with insecure attachment styles as people turn to food rather than relationships to manage their emotions [7–9]. For example, research indicates that those with eating disorders experience higher levels of attachment insecurity [10]; that conventional treatments may fail for those with eating disorders due to factors such as affect intolerance, interpersonal problems and clinical perfectionism [11] and that awareness of an individual’s attachment style may reduce drop-out rates from treatment and improve outcomes [11, 12].

Some research also indicates a role for attachment in obesity both in terms of its onset and management [13–15]. For example, some studies indicate an association between insecure maternal attachment and being overweight [16, 17] with anxious attachment being specifically linked with BMI possibly through the tendency to engage in disinhibited eating [18]. Similarly, Rommel et al. [19] identified an association between parental attachment and emotional awareness in obese patients, and Holland, Dallos and Olver [20] concluded from their qualitative study that complex, conflicted family relationships influence attachment styles which in turn lead to a reliance on food as a coping mechanism. In terms of attachment and weight loss, the findings are more mixed. For example, whilst weight loss has been linked with secure attachment [21] particularly, anxious attachment [15], Kieswetter et al. [22] found no relationship between attachment styles and weight loss. Further, a recent systematic review identified 13 papers exploring the links between attachment and obesity and concluded that whilst insecure attachment may be a contributory factor to weight gain the research on weight loss is limited [23]. The present study therefore explored the role of attachment styles in obesity. In particular, the study first explored the role of attachment styles in obesity by comparing attachment styles between those undergoing bariatric surgery to a matched control group of normal weight individuals, and second, explored the role of attachment styles in predicting weight loss post bariatric surgery at 1-year follow-up.

## Methods

### Design

The study involved both a cross sectional and cohort quantitative design and was embedded within a larger randomised controlled trial [24]. Measures of eating behaviour and health status were also assessed but recidivism rates were high so these are not included in this paper (*n* = 70 completed both baseline and follow-up measures; 35% response rate).

### Participants

Bariatric patients were recruited from a teaching hospital in the South East of England which offers a NHS-based bariatric service for obese patients with a BMI over 40. Inclusion criteria were aged over 18; had attended the bariatric clinic for a pre-assessment appointment; had been accepted for surgery by the bariatric team and had their funding for surgery agreed. Two hundred twelve patients were invited to participate. Of these 195 participants completed the baseline measures, 152 had surgery and 143 completed both attachment measures and had a BMI measure at 1 year follow-up. Surgery types were as follows: gastric bypass, *n* = 67; gastric sleeve, *n* = 73; gastric band, *n* = 2 and other =1.

For comparison, an opportunistic control group of normal weight (BMI of 18–30) individuals were simultaneously recruited using social media and asked to complete a measure of attachment and demographics. Three hundred sixty participants were initially recruited for the control group; these responses were screened, and 195 participants were selected to be matched as closely as possible to those in the bariatric group in relation to age, sex and height. Informed consent was obtained from all individual participants included in the study.

### Measures

Measures were completed by both the bariatric and control samples as follows. Reliability was assessed where appropriate using Cronbach’s alpha.

Both control and bariatric participants completed the measures listed below.

#### Demographics

Age, sex, weight and height. For the control group, height and weight were self report; for the bariatric group, they were collected during the pre-assessment appointment. The weight of the bariatric patients was also measured at 1-year follow-up.

#### Attachment

This was assessed using the Experiences in Close Relationships-Revised (ECR-R) Questionnaire [25]. The ECR-R is a 36 item measure to assess attachment-related anxiety (i.e. the extent to which people are insecure vs. secure about the extent to which their partner’s availability and responsiveness) and attachment-related avoidance (i.e. the extent to which people are uncomfortable being close to others vs. secure depending on others). Answers are given on a 7-point scale ranging from 1 “strongly disagree” to 7 “strongly agree”. The ECR-R has been shown to be a reliable measure with the most commonly cited estimate of reliability being 0.90 or higher [26]. For this study, reliability was acceptable (anxiety *α* = 0.93; attachment *α* = 0.86). The ECR-R has also shown to be valid and to be a highly stable indicator of attachment during a 3-week period and a significant predictor of social interaction emotions with a romantic partner, family and friends [26].

## Results

The data were analysed to explore differences in demographics and attachment styles between the control and bariatric samples using *t* tests and chi-square. The bariatric sample were then analysed in terms of their changes in weight and the role of baseline attachment in predicting changes weight post bariatric surgery using multiple regression analysis.

1. Differences between the control and bariatric samples (see Table 1)
  The results showed that the control and bariatric groups were comparable in terms of age, sex and height; whereas, those in the bariatric group showed a significantly higher weight and BMI. In addition, those in the bariatric group reported significantly higher levels of anxious attachment and significantly lower levels of avoidant attachment.
2. Changes in weight post bariatric surgery
  The mean change in BMI for all bariatric patients from baseline to 1-year follow-up was 12.62, SD = 4.7. No differences in change in BMI were found between men (*n* = 25, mean change = 13.11; SD) and women (*n* = 118; mean change = 12.5; SD = 4.22), (*t* = 0.54; *p* = 0.59; CI = −1.53–2.66) or between type of surgery in terms of gastric bypass (*n* = 67; mean change = 13.1, SD = 4.38) or sleeve (*n* = 73; mean change = 12.2; SD = 5.13), (*t* = 1.19, *p* = 0.24, CI = −0.65–2.56).
3. The role of attachment in predicting change in BMI post bariatric surgery
  Using the bariatrics sample (*n* = 143), multiple regression analysis showed that neither anxious attachment (*B* = 0.006, *p* = 0.9) nor avoidant attachment (*B* = −0.012, *p* = 0.89) predicted change in BMI by 1-year follow-up after bariatric surgery (adj *R* ^2^ = −0.01; *F* = 0.008, *p* = 0.9). The results also showed comparable results for those who had had a gastric bypass (adj *R* ^2^ = 0.007; *F* = 0.76; *p* = 0.5), and those who had had a sleeve (adj *R* ^2^ = −0.02; *F* = 0.4; *p* = 0.7).

**Table 1:** Participant demographics and attachment style by group (*n* = 390)

	Control sample (*n* = 195)	Bariatric sample (*n* = 195)	*χ* ^2^/t/p
Age	Mean = 38.33 SD = 11.04	Mean = 43.52 SD = 11.93	*t* = 2.81 *P* = 0.06 CI of the diff = −7.49 − −2.9
Sex	M = 42 (21.5%) F = 153 (78.5%)	M = 41 (21%) F = 154 (79%)	*χ* ^2^ = 0.02 *p* = 0.5 CI = 0.64–1.68
Height	Mean = 1.67 SD = 0.09	Mean = 1.67 SD = 0.09	*t* = −0.18 *p* = 0.9 CI of the diff = −0.19 – 0.16
Weight	Mean = 68.84 SD = 10.75	Mean = 129.4 SD = 25.81	*t* = −29.13 *p* = 0.0001 CI of the diff = −64.5 − −56.54
BMI	Mean = 24.47 SD = 2.9	Mean = 45.64 SD = 7.19	*t* = −35.9 *p* = 0.0001 CI of the diff = −22.3 − −20.1
Avoidant attachment	Mean = 3.27 SD = 1.15	Mean = 2.81 SD = 0.88	*t* = 4.44 *p* = 0.0001 CI of the diff = −0.81 − −0.54
Anxious attachment	Mean = 3.45 SD = 0.79	Mean = 4.13 SD = 0.58	*t* = −9.67 *p* = 0.001 CI of the diff =0.26–0.67

## Conclusion

Attachment theory suggests that insecure attachment may lead to the use of food to manage emotions [1–5]. It has therefore been argued that attachment styles may have a role to play in the development and management of obesity [13–20]. The results from the present study indicate differences between bariatric and normal weight participants supporting a role for attachment in the development of obesity. In particular, the bariatric patients reported higher anxious attachment and lower avoidant attachment. Given research emphasising the stability of attachment [1–5] which seems to be set in childhood and persist throughout the lifespan, these findings may reflect the use of food as a means to regulate emotions leading to weight gain. These findings support previous research highlighting a link between attachment and obesity [16–20] and indicate that it is insecure attachment per se which is of relevance rather than a specific subtype.

In terms of weight loss post surgery, the results indicated substantial change in BMI across the bariatric sample which was unrelated to gender or surgery type. Change in BMI post surgery was also found to be unrelated to attachment styles. This supports some previous research which has likewise found no impact of attachment on weight loss [22] but conflicts with others [15, 21]. There are several reasons for this. First, this may reflect that attachment is not a useful variable in this context and that other factors are at play which predict weight loss. Second, it may indicate that whilst attachment may play a role, other psychological variables such as dietary adherence, mood, self-esteem or self-efficacy which were not measured in the present study are stronger drivers. Finally, these findings may illustrate the uniformity of weight loss by 1-year due to the initial responses to surgery and that longer-term follow-ups are required if variability due to psychological factors are to be detected.

There are some problems with the present study that need to be addressed. First, although the study focused on the development of obesity, this involved a cross sectional comparison between an obese and non-obese sample rather than a longitudinal study exploring changes over time. However, given that attachment is developed in childhood, this would require a very long-term cohort study which has practical and financial implications. Further, much research emphasises attachment as a trait rather than a state suggesting that it is fixed from an early age, and therefore unlikely to change over the lifespan [1–5]. It is possible, however, that given the stigmatised nature of obesity, attachment may change for this population over their life course if they are confronted with negative responses from romantic partners. Further research could address changes in attachment in this population. Second, the study used a follow-up of only 1-year post surgery whereas much research indicates maximum variability and weight regain between 18 and 24 months [27]. This may explain the absence of an effect for attachment suggesting that longer-term follow-ups are required.

To conclude, the results from the present study indicate that whilst attachment style differed between the obese and non-obese samples, it was unrelated to weight loss following bariatric surgery. This has implications for understanding the role of early childhood experiences on weight gain and could be used as the basis for developing parenting programmes for those at risk of having overweight children. Future research could explore the longer-term impact of psychological variables on health outcomes following bariatric surgery.
